# Bees eavesdrop upon informative and persistent signal compounds in alarm pheromones

**DOI:** 10.1038/srep25693

**Published:** 2016-05-09

**Authors:** Zhengwei Wang, Ping Wen, Yufeng Qu, Shihao Dong, Jianjun Li, Ken Tan, James C. Nieh

**Affiliations:** 1 Key Laboratory of Tropical Forest Ecology, Xishuangbanna Tropical Botanical Garden, Chinese Academy of Science, Kunming, Yunnan Province, 650223 China; 2 Eastern Bee Research Institute, Yunnan Agricultural University, 650201 Kunming, China; 3 Division of Biological Sciences Section of Ecology, Behavior, and Evolution University of California, San Diego La Jolla, California, USA

## Abstract

Pollinators such as bees provide a critical ecosystem service that can be impaired by information about predation. We provide the first evidence for olfactory eavesdropping and avoidance of heterospecific alarm signals, alarm pheromones, at food sources in bees. We predicted that foragers could eavesdrop upon heterospecific alarm pheromones, and would detect and avoid conspicuous individual pheromone compounds, defined by abundance and their ability to persist. We show that *Apis cerana* foragers avoid the distinctive alarm pheromones of *A. dorsata* and *A. mellifera*, species that share the same floral resources and predators. We next examined responses to individual alarm pheromone compounds. *Apis cerana* foragers avoided isopentyl acetate (IPA), which is found in all three species and is the most abundant and volatile of the tested compounds. Interestingly, *A. cerana* also avoided an odor component, gamma-octanoic lactone (GOL), which is >150-fold less volatile than IPA. Chemical analyses confirmed that GOL is only present in *A. dorsata*, not in *A. cerana.* Electroantennogram (EAG) recordings revealed that *A. cerana* antennae are 10-fold more sensitive to GOL than to other tested compounds. Thus, the eavesdropping strategy is shaped by signal conspicuousness (abundance and commonality) and signal persistence (volatility).

Eavesdropping, or public information parasitism, is an intriguing, widespread phenomenon that structures how animals use information^1^ and how signals evolve^2^,^3^,^4^,^5^,^6^. Eavesdropping occurs when a signal, a feature that has been selected to convey information, is intercepted by unintended receivers^5^. For example, social bees eavesdrop upon heterospecific food-marking pheromones to find rewarding food resources^7^,^8^. These pheromones are chemical signals produced by specific glands and have evolved to provide food location information^9^. In contrast, bees can also detect cues, information that has not evolved to provide information in a given context, such as the cuticular hydrocarbon footprints produced by other bee species, and use this to avoid inflorescences that have already been depleted of nectar or to visit resources that are repeatedly rewarding^10^,^11^,^12^,^13^. Social bees can also avoid dangerous inflorescences where conspecifics were attacked and deposited alarm cues^14^. However, such cue detection, unlike eavesdropping, does not generally affect signal evolution because cues are not selected to convey information.

Signal evolution can be shaped by two general outcomes of eavesdropping: conflict (parasitism) or benefit (commensalism or mutualism). Conflict may occur over competition for limited resources^8^ and should favor information concealment by the signaler, though there are exceptions^7^. Benefit occurs when the signaler or eavesdropper gains and the other is not harmed (commensalism) or when both profit (mutualism). A classic example of eavesdropping mutualism occurs in bird mobbing calls that elicit multispecies attacks against a common predator^2^.

Here, we focus on eavesdropping that is either beneficial or neutral to signalers. For example, prey could avoid shared predators by eavesdropping to recognize interspecific warning signals^15^. Such eavesdropping is well documented in vertebrates^16^, but it is unclear if it occurs among pollinators. This is a question of broad ecological interest because pollinators play an important role in structuring food webs. Insects like bees pollinate approximately 75% of crop species and facilitate reproduction in 94% of wild flowering plants^17^. Predators can disrupt the plant-pollinator mutualism by deterring pollinator visitation, resulting in reduced seed^18^ and fruit production^19^,^20^. Thus, the ability of pollinators to detect information about predation has cascading ecosystem effects^21^. More broadly, the ability of prey to detect and avoid predators plays a major role in structuring ecosystems, in part by altering the spatio-temporal distribution of prey within a landscape^22^.

It is not known if pollinators can eavesdrop upon alarm signals to avoid predators. However, given that this behavior is either commensal or conveys mutual benefits to eavesdroppers and signalers, we expect alarm pheromone eavesdropping by foragers to be widespread among bees. Li *et al.*^23^ showed that *A. dorsata* can eavesdrop upon predator signals by detecting and avoiding the odor trail pheromone of weaver ants. Can foragers avoid predators by using social eavesdropping, intercepting social signals^5^? Highly social bees like honey bees produce alarm pheromones to coordinate colony defense^24^. This information is also useful in other contexts. Stingless bees, and honey bees can produce alarm pheromones when attacked at a food source and these pheromones elicit conspecific avoidance^25^,^26^.

Goodale and Nieh^27^ tested this question with bumble bees and honey bees, but found no evidence for such eavesdropping: bumble bees did not avoid honey bee alarm pheromone, a signal, though bumble bees avoided a cue that is a byproduct of predation, honey bee hemolymph odor. However, honey bees were introduced species in the tested environment. Eavesdropping is more likely to evolve when species are sympatric and share the same resources and predators over evolutionary time. In addition, eavesdropping is facilitated when signal elements are similar to the communication repertoire of the eavesdropper^2^ and provide conspicuous information. Eavesdropping should therefore occur among sympatric species of honey bees.

One interesting feature of olfactory signaling and eavesdropping is that odor signals, unlike acoustic signals, can linger in the environment^28^. Alarm pheromones can therefore provide immediate information and, through their less volatile components, convey information that is more lasting. Honey bees (*A. mellifera* and *A. dorsata*) respectively continued to avoid sting alarm pheromone for at least 20 min^27^ and 12 min^26^ after it was deposited on a food source. Thus, eavesdroppers could pay attention to odor abundance and to odor components that provide lasting warning information: the least volatile yet conspicuous elements of an alarm pheromone. The risk of predation does not need to be high for such information to have an impact. Although crab spiders only attack 4–11% of visiting bee pollinators^29^,^30^, such predators can reduce pollinator visitation rates by 36%^31^.

We therefore tested the hypothesis that pollinators can eavesdrop upon alarm signals and will pay specific attention to the most informative (abundant and persistent) signal components. We used two sympatric bee species, *Apis cerana* and *A. dorsata*, found throughout Southeast Asia^32^. At our field site in Xishuangbanna, China, *A. cerana* and *A. dorsata* naturally co-occur in dry seasons from December to July, until the rainy season begins and *A. dorsata* begin their annual migrations^33^. Our study was inspired by observations of these species jointly foraging on *Calliandra haematocephala* inflorescences and being preyed upon by a common predator, weaver ants (Fig. 1A), part of a guild of shared native predators^23^,^34^,^35^. *Apis mellifera* also occurred at this site, although it is a non-native species. We hypothesized that the *A. cerana* foragers would detect and avoid the alarm pheromones of *A. dorsata* and *A. mellifera*. The alarm pheromones of these three species share some compounds, but also contain distinctive compounds (Fig. 1C)^26^. Moreover, the most abundant *A. dorsata* alarm pheromone compounds span a >300-fold difference in volatility. To determine how volatility influences eavesdropping, we then tested forager responses to individual compounds. We predicted that *A. cerana* foragers would exhibit eavesdropping to *A. dorsata* and *A. mellifera* alarm pheromones, and would specifically detect and avoid conspicuous individual compounds, defined both by abundance and by decreased volatility.

## Results

We tested bee responses to natural sting pheromone and the following compounds: isopentyl acetate (IPA), 3-methyl-1-butanol (MB), isopentyl propionate (IP), octyl acetate (OA), gamma-octanoic lactone (GOL), (*E*)-2-decen-1-yl acetate (DA), and (*Z*)-11-eicosen-1-ol (EH).

### Foraging choices

**Figure 1:**
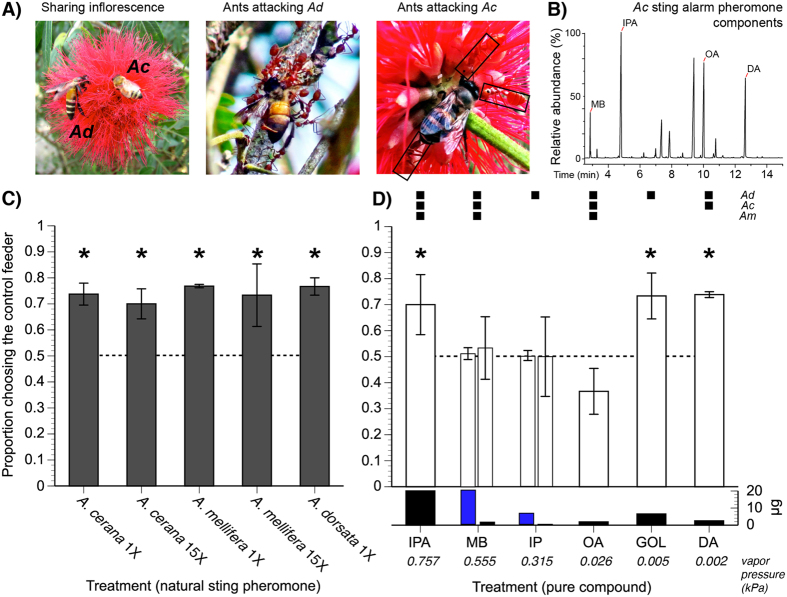
Behavioral responses of *A. cerana* (*Ac*) to *A. dorsata* (*Ad*) and *A. mellifera* (*Am*) sting alarm pheromones. (**A**) Images showing *Ac* and *Ad* sharing a *C. haematocephala* inflorescence at the field site, and being attacked by weaver ants (*Oecophylla smaragdina*) while foraging on these plants. The ants are outlined on the third image because they are similar in color to the inflorescence. (**B**) Chromatogram confirming *Ac* sting alarm pheromone components. Results of bioassays testing *Ac* forager avoidance of (**C**) sting alarm pheromone from different species of bees at different levels (1 or 15 bee equivalents = 1 or 15X) or (**D**) major sting pheromone components. To control for the possibility that bees did not avoid 1 *Ad* bee equivalent of MB or IP, we also tested their responses to higher levels of these compounds, which elicited clear antennal responses. The tested amount of each compound is shown below the bioassay results: black bars = 1 *Ad* equivalent and blue bars = higher levels. Compounds are arranged by decreasing volatility (lower vapor pressure = lower volatility). The dashed line gives the null expectation of no feeder preference. Asterisks denote treatments that elicited significant avoidance (*P* < 0.05, see Table 1). The mean ± 1 standard error is shown.

*Apis cerana* foragers significantly avoided the sting gland alarm pheromones of *A. cerana*, *A. mellifera*, and *A. dorsata* foragers at all tested levels (Table 1, Fig. 1C *P* ≤ 0.028). On average, 74 ± 2% of bees chose the control feeder (Fig. 1B). Bees did not exhibit greater avoidance to higher levels of sting gland pheromone (15X vs. 1X, Tukey’s HSD test, *P* > 0.05).

Our SPME-GC-MS analysis provided the first evidence that MB and DA are present in the sting alarm pheromone of *A. cerana* (Fig. 1B). We then tested the response of *A. cerana* foragers to *A. dorsata* alarm pheromone components.

*Apis cerana* foragers significantly avoided three of the alarm pheromone compounds (Fig. 1D): IPA, GOL, and DA (Table 1, *P* ≤ 0.01). There was no significant difference between avoidance of these three compounds and avoidance of natural pheromones (treatment effect: *F*_7,14_ = 0.10, *P* = 0.998, colony accounted for 1.4% of model variance) or between any of the pairwise comparisons of treatments that elicited avoidance (Tukey’s HSD test, *P* < 0.05). Foragers did not avoid MB or IP (Fig. 1D), even at levels that elicited strong antennal responses (see below).

### Antennal responses

**Figure 2:**
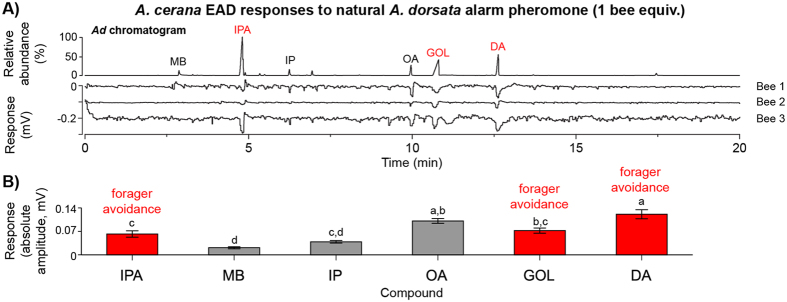
Antennal responses of *A. cerana* to natural *A. dorsata* alarm pheromone. (**A**) Representative electroantennograms and (**B**) average responses (means ± 1 standard error) from all bees to individual compounds presented at the levels found in natural *A. dorsata* alarm pheromone. Different letters indicate significant differences (Tukey’s HSD test, *P* < 0.05). Compounds that elicited forager avoidance (Fig. 1) are shown in red.

Our EAD analyses showed that *A. cer*ana foragers could detect and respond to multiple compounds in natural *A. dorsata* alarm pheromone (Fig. 2). Their responses varied according to the tested compound at its natural abundance level in *A. dorsata* sting alarm pheromone (*F*_5,40_ = 30.55, *P* < 0.0001). In particular, responses were stronger to compounds that elicited behavioral avoidance (IPA, GOL, and DA) in foraging bioassays than to MB, which did not elicit such avoidance (Fig. 2B, Tukey’s HSD test, *P* < 0.05).

**Figure 3:**
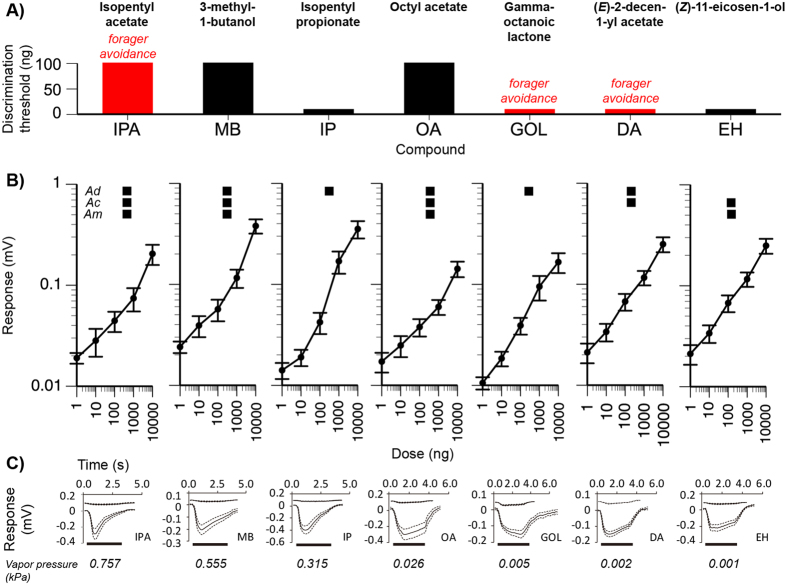
Antennal responses of *A. cerana* foragers to pure compounds found in sting gland pheromones. (**A**) The antennal discrimination threshold for each compound (lower value = greater sensitivity). Compounds that elicited forager avoidance (see Fig. 1) are indicated in red. (**B**) The dose-response curve (mean and standard errors, SE) for each compound. The bee species (*A. dorsata* = *Ad*, *A. cerana* = *Ac*, and *A. mellifera* = *Am*) corresponding to these compounds are indicated. (**C**) Average EAG response curves calculated from all responses to 10,000 ng of the tested compound. Mean responses (solid line) with SE (dashed lines) are shown. The horizontal bar indicates the stimulus duration. Control responses (mean and SE) are shown above the response to each compound.

We next tested *A. cerana* forager responses (EAG) to pure compounds over the same range of levels to determine bee antennal discrimination thresholds, defined as the minimum dose that elicited a significantly different EAG response as compared to the EAG response to the blank control (Fig. 3A). As in the EAD experiment, *A. cerana* forager antennae responded to all tested compounds including GOL, which is not found in conspecific alarm pheromone (Fig. 3B,C). In our overall model, there was a significant effect of dose (*F*_5,385_ = 405.16, *P* < 0.0001) and the interaction dose*compound (*F*_30,385_ = 2.55, *P* < 0.0001) because the dose response curves had different slopes (Fig. 3B). Colony accounted for 7.0% of model variance.

For IP, GOL, DA, and EH, the discrimination threshold was 10 ng (Dunnett’s Method, *P* ≤ 0.003). For IPA, MB, and OA, the discrimination threshold was 100 ng (Dunnett’s Method, *P *≤* *0.01). Thus, *A. cerana* antennae were approximately 10-fold more responsive to IP, GOL, DA, and EH as compared to all other tested compounds (Fig. 3A).

## Discussion

The habitat of *A. cerana* overlaps with the native heterospecific *A. dorsata* and the introduced *A. mellifera*. *Apis cerana* should therefore benefit from avoiding its own alarm pheromone and eavesdropping upon heterospecific alarm pheromones to reduce foraging predation risks. Our results confirm this prediction. *Apis cerana* foragers avoided the sting alarm pheromone of its own species and heterospecifics at a food source. Moreover, *A. cerana* foragers avoided GOL, a component of *A. dorsata* alarm pheromone that is not found in *A. cerana* alarm pheromone, and thereby exhibited classic olfactory eavesdropping. This avoidance was not due to neophobia. Foragers did not avoid MB or IP, even at much higher, supra-threshold levels (Fig. 1D). Foragers also did not avoid OA, which shares a similar ester structure with GOL (Fig. 1D). Synthetic alarm pheromone components (IPA, GOL, and DA) did not elicit significantly different avoidance as compared to natural alarm pheromones. Finally, *Apis cerana* avoidance of natural alarm pheromone did not increase with elevated pheromone levels. Foragers equally avoided 15 bee and 1 bee equivalents of *A. cerana* or *A. mellifera* sting pheromone (Fig. 1C), suggesting that the avoidance response depended upon the alarm pheromone exceeding a threshold level.

The feeder bioassays tested each component individually because we wished to focus on eavesdropping. DA, IP, and GOL are semiochemicals found in *A. dorsata* but not *A. cerana*, and we therefore measured the responses of *A. cerana* to these individual compounds. However, future assays testing the effects of different compound combinations would be informative.

Our EAD results demonstrated that *A. cerana* antennae respond to natural *A. dorsata* alarm pheromone (Fig. 2). The EAG results (Fig. 3) showed overlap between antennal discrimination thresholds and behavioral avoidance. In foraging assays, *A*. *cerana* significantly avoided GOL and DA and their antennae had 10-fold lower discrimination thresholds for these compounds. However, the correspondence between behavior and antennal responses was not complete because antennae were also quite sensitive to IP (which did not elicit avoidance) and less sensitive to IPA (which elicited avoidance). This result is not surprising given that other brain regions—olfactory lobes and mushroom bodies—play a role in olfactory processing and behavioral responses^36^.

Although OA is found in *A. cerana* alarm pheromone, foragers did not avoid this compound (Fig. 1C). OA occurs in the alarm pheromone of *A. mellifera* and attracts workers near the nest entrance^37^. The response of *A. cerana* to OA at the nest entrance is unknown, but OA did not repel foragers at a food source. The reason for this non-avoidance is unclear, although OA was tested at a lower level than the compounds (IPA, GOL, and DA) that elicited avoidance. To test eavesdropping, we used the average amount of each compound found in a single *A. dorsata* sting gland. Thus, IPA (20.0 μg), GOL (6.5 μg), and DA (2.5 μg) were more abundant than OA (1.9 μg). In addition, different alarm pheromone components can elicit different responses^38^.

Receivers should exploit the most abundant and informative signals in their environment. Our results support this hypothesis. IPA is the most abundant compound in *A. cerana*, *A. dorsata* and *A. mellifera* alarm pheromones^26^,^39^. Although GOL and DA are less abundant than IPA, they are the second and third most abundant compounds and are, on average, >128-fold less volatile than IPA. Their use therefore illustrates another aspect of olfactory conspicuousness: information persistence. Individuals should use the most conspicuous information^3^, but olfactory conspicuousness changes with time. Although IPA is produced in the greatest quantity, GOL and DA should persist the longest because they are the least volatile.

In general, eavesdropping is more likely to evolve when unintended receivers can detect components of a signal, when such signals are similar to those used by the eavesdropper, or both are true. Both IPA and DA are found in the sting alarm pheromones of *A. cerana* and *A. dorsata*, and thus it is not surprising that *A. cerana* avoided these components. However, GOL is not found in *A. cerana*^40^ (Fig. 1B), and no compounds with similar lactone structures have yet been reported in *A. cerana*. Given that *A. cerana* and *A. dorsata* are sympatric, have co-existed over evolutionary time^41^, and share common floral predators^23^,^34^,^35^, an innate, eavesdropping alarm response to GOL could have evolved.

Demonstrating that this ability is innate will require further study because bees could have learned these compounds. At our field site, *A. cerana* and *A. dorsata* and *A. mellifera* were present at the same time on natural floral resources. Because *Apis cerana* is sympatric with *A. dorsata* and allopatric with *A. mellifera*, we predict that *A. cerana* foragers could have an innate aversion to alarm components found only in *A. dorsata* but not to components found only in *A. mellifera.* This is a testable hypothesis: *A. mellifera* alarm pheromone contains multiple components not found in *A. ceranae* alarm pheromone^26^.

An alternative explanation is that GOL was an allomone, a chemical signal designed to manipulate receiver behavior for the benefit of the signal sender^42^. Has *A. dorsata* evolved GOL to repel *A. cerana* and thereby avoid fights? This is highly unlikely because GOL is a key component of *A. dorsata* alarm pheromone and elicits conspecific alarm. In fact, GOL repels *A. dorsata* foragers from inflorescences where conspecifics are attacked^26^. Interference competition has been reported between *A. dorsata* and *A. cerana* at an artificial feeder^43^ but not at natural food sources. At our field site, both bee species shared inflorescences (Fig. 1A) and foraged next to each other without repulsion or aggression. Moreover, both species share common predators^23^,^34^,^35^.

In summary, public information about predators contributes to an information web that helps to shape food and interaction webs^44^. This public information plays a key role in how predators influence prey^22^. Our results suggest that we should consider a broad olfactory landscape that includes pollinator cues and social signals. An interesting question is how temporal aspects of this information shape predator avoidance. Because olfactory information provides a clock, data about when an event occurred, bees can eavesdrop upon past events to influence their current decisions to forage. Thus, olfactory eavesdropping can take advantage of highly specific information: a signal at a point in space and past time. Whether eavesdropping pollinators take advantage of this time component to finely modulate their behavior remains to be determined, but increases the potential richness of this information web.

## Materials and Methods

### Bee alarm pheromone extracts

We conducted our experiments at Yunnan Agricultural University, Kunming, China. To obtain sting alarm pheromone, we used an aspirator to carefully capture 15 guard bees from the entrances of three colonies of *A. cerana* and *A. mellifera* (45 bees per species). *Apis dorsata* foragers were collected from flowers at the Xishuangbanna Tropic Botanic Garden (Menglun, China). We immediately froze the bees after capturing them and subsequently thawed them to dissect out the intact sting gland with clean forceps. To dissolve the fatty acid membrane of the sting gland, the glands were placed into separate, clean 2 ml amber glass vials with 300 μl hexane at room temperature for 30 min. These extracts were then stored in a 4 °C refrigerator for subsequent *A. cerana* forager choice bioassays. To determine the effect of sting pheromone quantity, we tested *A. cerana* responses to 1 or 15 bee equivalents (1X or 15X) of *A. cerana* or *A. mellifera* sting alarm pheromone extracts or 1X of *A. dorsata* sting pheromone extract. We only used 1X of *A. dorsata* sting pheromone extract because *A. cerana* showed the same level of avoidance to 1X and 15X of *A. cerana* or *A. mellifera* sting pheromone extracts. For each trial, we used an extract obtained from the pooled sting alarm pheromone of a different set of bees.

We used guard bees to prepare extracts because, in *A. mellifera*, the sting gland pheromones of guards and foragers have similar levels of the major component, isopentyl acetate, and differ somewhat in the levels of minor components^45^. Abundance of natural *A. mellifera* or *A. cerana* sting pheromone (1X versus 15X) did not significantly affect our results (Table 1), and thus we expect that using sting alarm pheromone from foragers would have yielded similar results.

We also tested the response of *A. cerana* foragers to the major identified compounds in the sting glands of *A. dorsata*. In each bioassay, we tested a biologically relevant amount, the mean quantity of compound found in a single, filled *A. dorsata* forager sting gland: 20.0 μg of isopentyl acetate (IPA), 1.8 μg of 3-methyl-1-butanol (MB), 0.5 μg of isopentyl propionate (IP), 1.9 μg of octyl acetate (OA), 6.5 μg of gamma-octanoic lactone (GOL), and 2.5 μg of DA (*E*)-2-decen-1-yl acetate^26^.

To control for potential avoidance of novel odors (neophobia), we subsequently tested responses to supra-threshold levels of MB (20 μg) and IP (6.5 μg) based upon discrimination threshold data. MB and IPA share the same discrimination threshold and have a similar volatility (Fig. 3B), and foragers strongly avoided IPA at a level of 20 μg (Fig. 1C). IP and GOL share the same discrimination threshold and have a similar volatility (Fig. 3B). Foragers strongly avoided GOL at a level of 6.5 μg (Fig. 1C).

The DA was synthesized by J. Millar^26^. All other compounds listed above were purchased from the Aladdin Reagent Database Inc. (Shanghai, China). All three bee species share MB, IPA and OA in their alarm pheromones (Table 1, Fig. 1). However, *A. cerana* sting alarm pheromone does not contain IP, or GOL^26^,^40^.

To confirm the sting gland components of *A. cerana*, we used vials to capture nine foragers from three colonies (three foragers per colony), cold anesthesized them, and carefully dissected out their sting glands. Each dissected sting gland was extracted for 30 min using headspace solid phase microextraction (SPME) with a 65 μm PDMS/DVB fiber (Supelco, CA) in a 2 ml vial (Agilent, US). The samples were subjected to GC analysis as described in GC-EAD below and GC-MS analysis as described by Li *et al.*^23^. Compounds were verified against pure chemical standards. This analysis confirmed the known components (IPA and OA)^26^,^40^ and showed two components (MB and DA) not previously identified in this species (Fig. 1B).

### Behavioral tests

We presented *A. cerana* foragers with a two-choice feeder bioassay to test the aversive effect of alarm pheromones from different species (*A. cerana*, *A. mellifera*, and *A. dorsata*) and alarm pheromone components (IPA, MB, IP, OA, GOL, and DA). Three different *A. cerana* colonies were sequentially used. Each day, we performed only two trials, with a minimum time between trials of 3 h. Each olfactory treatment was replicated in three trials, with the exception of testing responses to *A. dorsata* alarm pheromone (six replicate trials). The order of olfactory treatments presented on any given day was randomized.

We trained *A. cerana* foragers to a feeder containing 50 ml of 1.5 M unscented sucrose solution and located 120 m from the focal colony. The feeder consisted of an inverted 70 ml glass vial with 18 holes (each 3 mm in diameter) drilled into the lid. The feeder was placed on a blue card to facilitate forager training and recognition. Once 10 foragers were trained from the focal colony, we removed the training feeder and placed two identical feeders, each containing 50 ml of unscented 2.0 M sucrose solution and separated by 50 cm, located 2 m away from the training feeder position. We placed each of these feeders on top of a 12.5 cm diameter filter paper inside a 14 cm diameter glass petri dish.

At the beginning of the trial, we pipetted out four equidistant dots (2.5 μl each) of odor extract onto the filter paper. In total, this amount corresponded to half the mean quantity of the tested compound in an *A. dorsata* forager sting gland. The control feeder had four dots of the solvent, pure hexane (also 2.5 μl/dot). Each trial lasted 20 min. By using headspace solid phase microextraction gas chromatography (HD-SPME-GC) analysis, we found that 90% of these chemicals would evaporate within 10 min under the test conditions at our field site. We therefore added a second dose of test compound to ensure that enough remained to be detectable. Because we used a 0.5 bee-equivalent each 10 min, we applied a total of 1 bee-equivalent over the 20 min trial.

For trials testing potential neophobia (see above), we used larger quantities of MB and IP (half also applied each 10 min) to ensure that these compounds were detectable by foragers. We swapped the feeder locations each 5 min to reduce the effects of potential site bias. The filter papers were replaced by new ones every trial and all feeders and petri dishes were carefully cleaned between trials to remove odors.

To exclude the possibility of bees being influenced by the choices of other bees, we only counted choices made in the absence of other bees foraging near the feeders. Each bee choice was counted only once since the bees that landed on feeder were immediately captured with an aspirator. Once a bee landed, it was immediately captured with an aspirator and then the landing spot was cleaned with a cotton swab soaked in 75% ethanol to remove potential bee-deposited odors. Bees only landed on the plastic lid of the feeder. At the end of each trial, all captured bees were frozen. We usually tested 10 bees per trial (30 bees per treatment because we replicated with three colonies), but in some cases, there was recruitment to the feeder and more bees arrived than were trained. On average, we tested 13.3 ± 5.1 bees per trial (1.7 ± 0.4 min between bees).

### Coupled gas chromatography-electroantennogram detection (GC-EAD) analysis

To determine if *A. cerana* foragers can detect the chief compounds found in natural *A. dorsata* alarm pheromone, we conducted coupled gas chromatography-electroantennogram detection (GC-EAD) analyses. Antennae of *Apis cerana* foragers were exposed to the GC-separated components of natural *A. dorsata* alarm pheromone (the head-space SPME extracts of alarm pheromone released by attacked bees inside 5 ml glass vials, methods of Li *et al.*^26^). We used a HP-7890B GC (Agilent, US) and desorbed the SPME extracts in a splitless injection port at 250 °C. We used an HP-5 column (30 m × 320 μm × 0.25 μm, Agilent, US) with nitrogen (2 ml/min) as the carrier gas. The oven temperature was 50 °C for 2 min and then increased at 10 °C/min to 230 °C. We used a Flame Ionization Detector (FID) heated to 300 °C to detect all compounds. The electroantennogram (EAG) system described below was connected to this GC system with a custom, 40 cm heated (250 °C) transfer line. To maximize the amount of pheromone reaching the antennae, we did not use a capillary column splitter. The EAD signals and FID signals were separately recorded. We replicated EADs with nine *A. cerana* foragers from three colonies, each bee tested with the alarm pheromone from a different *A. dorsata* forager (total of nine *A. dorsata* foragers).

### Electroantennogram (EAG) analysis

To determine if *A. cerana* foragers could detect the pure olfactory compounds in sting alarm pheromone and to characterize their antennal responses, we used electroantennogram (EAG) analysis and tested responses to the same compounds: IPA, MB, IP, OA, GOL, and DA. In our EAG tests, we also tested a component of *A. cerana* sting pheromone, (*Z*)-11-eicosen-1-ol (EH), which is not found in *A. dorsata* sting pheromone^26^. EH cannot be purchased and was therefore not used for the foraging bioassays. However, we were able to purify EH in sufficient quantities for EAG testing. EH was purified from the sting extracts of 15 *A. cerana* foragers using silica chromatography^46^ and then quantified with GC-MS, using eicosan-1-ol (Aladdin, CN) as an internal standard.

In preliminary trials, we compared the responses of freshly dissected antennae and entire bee heads and found no differences. However, our setup was quite sensitive and could detect signals from antennal muscle movements in intact heads. We therefore chose to use cut antennae, randomly selecting the left or right antennae per bee because preliminary tests found no differences in the responses of left or right antennae.

To record the antennal response, we first carefully captured an *A. cerana* forager, chilled it briefly to reduce its motion, cut off both antennae, and placed each antenna between glass electrodes filled with insect Ringer’s solution^47^. Each antenna was placed 1 cm away from the outlet of a PTFE tube (1 cm inner diameter, 15 cm long) that provided the test odor by combining clean (500 mL active charcoal filtered) and wet (distilled water, 90% RH) 15 ml/s continuous air flows and pre-filtered and wet 5 ml/s pulsed air flows (90% RH) with the test odor. All measurements were conducted at 25 °C. For each stimulation, pulsed odor air flow was delivered into the odor pipette for 3 s, mixing into the continuous flow. To record the antennal responses, we used a custom stimulus controller, a modified EAG amplifier (Syntech, NL, but modified to increase sensitivity) outputting a signal into a HP34405A Digital Multi Meter (Agilent, USA) and BenchVue software (Keysight, USA) running on a PC.

For each compound, we measured the antennal response (voltage amplitude difference between the baseline and the peak) of 12 different bees (four bees per colony from three different colonies). The response of each bee was tested to six different doses: 0 ng (control) 1 ng, 10 ng, 100 ng, 1000 ng, and 10,000 ng in an ascending dose sequence. We waited at least 30 s between odor presentations to provide sufficient recovery time. In a preliminary experiment, we tested bee responses to GOL (intermediate in volatility among our tested compounds) at 1000 ng (above the bee discrimination thresholds for all tested compounds) and found that a 10 s time between presentations elicited responses that were not significantly different (*F*_1,9_ = 0.52, *P* = 0.49, 12 bees from three colonies). In these preliminary tests, we tested for an effect of randomizing odor dose presentation, but found no such effect. We therefore used ascending doses because this simplified the experiment.

All compounds were dissolved to achieve the correct dose in 1 μl of dichloromethane (DCM, Aladdin, CN). In all tests, we first measured bee responses (negligible) to 1 μl of pure DCM (see control responses in Fig. 3C). For highly volatile compounds (IPA, MB, and IP), the dichloromethane solution was directly pipetted onto the paper strip pre-positioned inside a 2 ml odor pipette. Bees may therefore have responded to both the DCM and the test compound. For these compounds, we subtracted the DCM reading from the first antennal response to the test compound. For the less volatile compounds (OA, GOL, DA and EH), we placed the solution on a paper strip and allowed it to evaporate for 10 s before placing the paper into the odor pipette. For all compounds, we averaged the first three responses to reduce noise.

### Statistics

To analyze our feeder choice data, we used Chi-square tests (Microsoft Excel v14.5) to determine if bees favored the control feeder over the experimental feeder. In these tests, our null expectation was that bees would exhibit no choice for either feeder. We also ran an Analysis of Variance (ANOVA) with a REML algorithm to test for differences between bee avoidance of the different olfactory treatments (colony included as a random effect) and made all pairwise comparisons with Tukey’s Honestly Significant Difference (HSD) tests to control the overall error rate^48^.

To test the olfactory ability of bees to discriminate single compounds (log transformed EAG response), we used a Repeated Measures ANOVA with bee identity nested within treatment (compound type, a fixed factor) and compound dose (fixed factor) as the repeated measure. Colony was included as a random factor. To compare EAG responses to each compound, we then ran a separate ANOVA at each dose level. We define the discrimination threshold as the minimum dose that elicited a significantly different EAG response from the control. To determine the discrimination threshold, we ran a Univariate Repeated Measures analysis (with bee identity as the repeated measure) and used Dunnett’s Method to compare all doses with the control. To analyze the EAD results, we also ran a Univariate Repeated Measures analysis with bee identity as the repeated measure. The ANOVA models met parametric assumptions as determined by residuals analysis. We used JMP Pro v11.2 statistical software. In our text, we report the mean ± 1 standard deviation. All data in this paper are available at the Dryad Digital Repository (http://dx.doi.org/10.5061/dryad.n53d0).

### Ethics

This study used the minimum number of animals necessary for robust data. The species used are not endangered or protected.

## Additional Information

**How to cite this article**: Wang, Z. *et al.* Bees eavesdrop upon informative and persistent signal compounds in alarm pheromones. *Sci. Rep.* **6**, 25693; doi: 10.1038/srep25693 (2016).

## Figures and Tables

**Table 1:** Summary of Chi-square tests (all have 1 d.f.) conducted to test bee avoidance natural sting gland alarm pheromone from different species of bees at different levels (1 or 15 bee equivalents = 1 or 15X) and pure synthetic compounds found at high levels in the sting alarm pheromone *A. dorsata*: isopentyl acetate (IPA), 3-methyl-1-butanol (MB), octyl acetate (OA), (*E*)-2-decen-1-yl acetate (DA), isopentyl propionate (IP), and gamma-octanoic lactone (GOL).

Treatment	No. of bees choosing				
	Test compound	Solvent control	% choosing control	Chi-square statistic	*P*
*A. cerana* 1X	14	38	73%	11.08	0.0009
*A. cerana* 15X	9	21	70%	4.80	0.0285
*A. mellifera* 1X	17	57	77%	21.62	<0.0001
*A. mellifera* 15X	8	22	73%	6.53	0.0106
*A. dorsata* 1X	11	64	85%	37.45	<0.0001
IPA _*Ad, Ac, Am*_	9	21	70%	4.80	0.0285
MB _*Ad, Ac, Am*_(1.8 μg*)	14	16	53%	0.13	0.7150
MB _*Ad, Ac, Am*_(20 μg**)	19	28	61%	1.72	0.1893
OA _*Ad, Ac, Am*_	19	11	37%	2.13	0.1441
DA _*Ad, Ac*_	15	43	74%	13.52	0.0002
IP _*Ad*_(0.5 μg*)	15	15	50%	0.00	1.0000
IP _*Ad*_(6.5 μg**)	23	23	50%	0.00	1.0000
GOL _*Ad*_	8	22	73%	6.53	0.0106

Subscripts indicate if these compounds are found in *A. cerana* (*Ac*), *A. dorsata (Ad*) or *A. mellifera* (*Am*). Compounds are ordered by species groupings. *Level in 1 *Ad* bee equivalent. **Higher, supra-threshold level used to test for neophobia.
